# Genome-wide analyses of platinum-induced ototoxicity in childhood cancer patients: Results of GO-CAT and United Kingdom MAGIC consortia

**DOI:** 10.3389/fphar.2022.980309

**Published:** 2023-01-09

**Authors:** Evelien G. E. Hurkmans, Marije J. Klumpers, Cinzia Dello Russo, Ward De Witte, Henk-Jan Guchelaar, Hans Gelderblom, Anne-Marie Cleton-Jansen, Sita H. Vermeulen, Suzanne Kaal, Winette T. A. van der Graaf, Uta Flucke, Corrie E. M. Gidding, Hendrik W. B. Schreuder, Eveline S. J. M. de Bont, Huib N. Caron, Giovanna Gattuso, Elisabetta Schiavello, Monica Terenziani, Maura Massimino, Geoff McCowage, Sumanth Nagabushan, Anuja Limaye, Victoria Rose, Daniel Catchpoole, Andrea L. Jorgensen, Christopher Barton, Lucy Delaney, Daniel B. Hawcutt, Munir Pirmohamed, Barry Pizer, Marieke J. H. Coenen, D. Maroeska W. M. te Loo

**Affiliations:** ^1^ Department of Human Genetics, Radboud University Medical Center, Nijmegen, Netherlands; ^2^ Department of Pediatrics, Radboud University Medical Center, Nijmegen, Netherlands; ^3^ Department of Pharmacology and Therapeutics, Institute of Systems, Molecular and Integrative Biology (ISMIB), University of Liverpool, Liverpool, United Kingdom; ^4^ Department of Healthcare Surveillance and Bioethics, Section of Pharmacology, Università Cattolica del Sacro Cuore-Fondazione Policlinico Universitario A. Gemelli IRCCS, Rome, Italy; ^5^ Department of Clinical Pharmacy & Toxicology, Leiden University Medical Center, Leiden, Netherlands; ^6^ Department of Medical Oncology, Leiden University Medical Center, Leiden, Netherlands; ^7^ Department of Pathology, Leiden University Medical Center, Leiden, Netherlands; ^8^ Department for Health Evidence, Radboud University Medical Center, Nijmegen, Netherlands; ^9^ Department of Medical Oncology, Radboud University Medical Center, Nijmegen, Netherlands; ^10^ Department of Medical Oncology, Netherlands Cancer Institute, Amsterdam, Netherlands; ^11^ Department of Pathology, Radboud University Medical Center, Nijmegen, Netherlands; ^12^ Princess Maxima Center for Pediatric Oncology, Utrecht, Netherlands; ^13^ Department of Orthopedics, Radboud University Medical Center, Nijmegen, Netherlands; ^14^ Department of Pediatrics, Beatrix Children’s Hospital, University Medical Center Groningen, Groningen, Netherlands; ^15^ Department of Pediatrics, Amsterdam University Medical Centers, Emma Children’s Hospital, Amsterdam, Netherlands; ^16^ Pediatric Oncology Unit, Fondazione IRCCS Istituto Nazionale dei Tumori, Milan, Italy; ^17^ Cancer Centre for Children, The Children’s Hospital at Westmead, Sydney, NSW, Australia; ^18^ Discipline of Child and Adolescent Health, University of Sydney, Sydney, NSW, Australia; ^19^ Department of Audiology, The Children’s Hospital at Westmead, Sydney, NSW, Australia; ^20^ Department of Neuro-Otology, Royal Prince Alfred Hospital, University of Sydney, Sydney, NSW, Australia; ^21^ Children’s Cancer Research Unit, The Children’s Hospital at Westmead, Sydney, NSW, Australia; ^22^ Department of Health Data Science, University of Liverpool, Liverpool, United Kingdom; ^23^ Department of Women’s and Children’s Health, University of Liverpool, Liverpool, United Kingdom; ^24^ NIHR Alder Hey Clinical Research Facility, Alder Hey Children’s Hospital, Liverpool, United Kingdom; ^25^ Department of Pharmacology and Therapeutics, University of Liverpool, Liverpool, United Kingdom; ^26^ Department of Pediatric Oncology, Alder Hey Children’s Hospital, Liverpool, United Kingdom

**Keywords:** childhood cancer, cisplatin, carboplatin, ototoxicity, GWAS, *TSPAN5*

## Abstract

Hearing loss (ototoxicity) is a major adverse effect of cisplatin and carboplatin chemotherapy. The aim of this study is to identify novel genetic variants that play a role in platinum-induced ototoxicity. Therefore, a genome-wide association study was performed in the Genetics of Childhood Cancer Treatment (GO-CAT) cohort (n = 261) and the United Kingdom Molecular Genetics of Adverse Drug Reactions in Children Study (United Kingdom MAGIC) cohort (n = 248). Results of both cohorts were combined in a meta-analysis. In primary analysis, patients with SIOP Boston Ototoxicity Scale grade ≥1 were considered cases, and patients with grade 0 were controls. Variants with a *p*-value <10^−5^ were replicated in previously published data by the PanCareLIFE cohort (n = 390). No genome-wide significant associations were found, but variants in *TSPAN5, RBBP4P5, AC010090.1* and *RNU6-38P* were suggestively associated with platinum-induced ototoxicity. The lowest *p*-value was found for rs7671702 in *TSPAN5* (odds ratio 2.0 (95% confidence interval 1.5–2.7), *p*-value 5.0 × 10^−7^). None of the associations were significant in the replication cohort, although the effect directions were consistent among all cohorts. Validation and functional understanding of these genetic variants could lead to more insights in the development of platinum-induced ototoxicity.

## 1 Introduction

Cisplatin and carboplatin are used as cornerstone anti-neoplastic treatments in many malignancies. Despite their anti-tumor effects, ototoxicity (or hearing loss) is a major adverse effect. Platinum-induced ototoxicity is generally irreversible and occurs in 42%–67% of patients treated with cisplatin (Berg et al., 1999; Stohr et al., 2005; Brock et al., 2012; Yancey et al., 2012) and in up to 20% of patients after carboplatin treatment (Qaddoumi et al., 2012; Clemens et al., 2017). Severe ototoxicity during chemotherapy treatment may lead to dose reduction of the platinum compound to prevent further ototoxicity, which has the inherent risk of reduced anti-tumor effect. In addition, ototoxicity has a negative effect on quality of life, especially in children treated for cancer. It has been shown that these children are at increased risk of learning and reading problems and psychosocial difficulties (Gurney et al., 2007; Olivier et al., 2019).

Clinical risk factors for platinum-induced ototoxicity include co-treatment with other ototoxic drugs such as aminoglycosides or furosemide, cumulative dose and infusion time of platinum treatment, cranial irradiation, young age and male sex (Langer et al., 2013). These risk factors can partly explain the interindividual differences in the development of platinum-induced ototoxicity, although genetic risk factors are also hypothesized to play a role. For example, Xu *et al.* found a common variant in the *ACYP2* gene (rs1872328) to be associated with cisplatin-induced ototoxicity (Xu et al., 2015). This finding has been replicated multiple times and meta-analysis showed an almost 4-times increased ototoxicity risk for patients carrying the G-allele (Thiesen et al., 2017; Clemens et al., 2020). Also, genetic variants in *TCERG1L, SLC22A2, WFS1, OTOS, ABCC3* and others have been suggested to play a role in cisplatin-induced ototoxicity (Pussegoda et al., 2013; Wheeler et al., 2017; Drogemoller et al., 2018; El Charif et al., 2019; Langer et al., 2020; Meijer et al., 2021). However, often due to conflicting results between studies, no widely accepted treatment protocols are in place for pharmacogenetic testing in clinical practice in order to identify patients at increased risk of platinum-induced ototoxicity (Langer et al., 2020; Meijer et al., 2021).

When comparing children to adults, other genetic variants may play a role in platinum-induced ototoxicity, e.g., due to increased sensitivity of the developing cochlea, and differences in gene expression levels (Drogemoller et al., 2019). Thereby, pharmacogenetic findings in adults cannot always be directly translated to children, underlining the need for studies in pediatric cohorts. As pediatric cancer is relatively rare, an international collaboration between the Genetics of Childhood Cancer Treatment (GO-CAT) consortium and United Kingdom Molecular Genetics of Adverse Drug Reactions in Children (MAGIC) study was initiated to establish an international cohort that is sufficient to perform statistically meaningful analyses. A genome-wide association study (GWAS) was performed in patients treated with these platinum agents from these childhood cancer cohorts. The recently published GWAS by the PanCareLIFE consortium served as a replication cohort (Meijer et al., 2021). The aim of the study was to identify novel genetic markers that contribute to prediction of the occurrence of platinum-induced ototoxicity and provide insights into its biological mechanisms.

## 2 Methods

### 2.1 Patients and treatment

The discovery study was a meta-analysis of GWASs of two patient cohorts; the GO-CAT cohort and the United Kingdom MAGIC study cohort. The GO-CAT cohort is a multinational retrospective cohort of pediatric cancer patients, treated between 1975 and 2020. Participating centers included Radboud university medical center (Nijmegen, Netherlands), University Medical Center of Groningen (Groningen, Netherlands), Leiden University Medical Center (Leiden, Netherlands), Academic Medical Center (Amsterdam, Netherlands), Fondazione IRCCS Istituto Nazionale Tumori (Milan, Italy) and The Children’s Hospital at Westmead (Sydney, Australia). The majority of this cohort was platinum-treated, and of a subset, genetic material was available sufficient for genome-wide genotyping. The United Kingdom MAGIC study cohort was a retrospective cohort of platinum-treated pediatric cancer patients recruited between January 2012 and March 2018 at eight United Kingdom sites: Alder Hey Children’s Hospital (Liverpool, United Kingdom), Leeds General Infirmary (Leeds, United Kingdom), Royal Manchester Children’s Hospital (Manchester United Kingdom), Great Ormond Street Hospital NHS Trust (London, United Kingdom), Nottingham University Hospitals NHS Trust (Nottingham, United Kingdom), Leicester Royal Infirmary NHS Trust (Leicester, United Kingdom), Newcastle Hospitals NHS Trust (Newcastle, Uinted Kingdom), and York Hill Hospital (Glasgow, United Kingdom) (Thiesen et al., 2017). These studies were approved by all local ethics committees. Written informed consent was obtained of all included patients that were alive at the moment of inclusion and/or their parents or legal guardians if applicable.

Inclusion criteria for this study were: 1) Patients were diagnosed with a tumor (with or without metastases), which was histologically proven or, if no tumor material had been investigated, confirmed by imaging. 2) Patients received primary chemotherapeutic treatment including a platinum agent. Patients from the GO-CAT cohort were treated with cisplatin or carboplatin, whereas patients from the United Kingdom MAGIC cohort were primarily treated with cisplatin. Exact treatment regimens depended on tumor type and local treatment protocols. 3) Availability of material for DNA isolation or genotyping data with genome-wide coverage, and passed genotyping quality control (Section 2.3). 4) Well-documented patient data were collected concerning baseline characteristics and treatment to establish clinical factors with potential impact on ototoxicity (e.g., age, platinum dose and concomitant use of other ototoxic drugs). Data regarding cranial or craniospinal irradiation was important since it has a known impact on hearing. Audiograms were collected at baseline, during chemotherapy (if available) and (at least one) during follow-up for ototoxicity assessment. Pre-existing clinically relevant hearing loss at baseline, being >20 dB hearing loss at any frequency, either unilateral or bilateral, was an exclusion criterium. Full inclusion and exclusion criteria for MAGIC have been published previously (Thiesen et al., 2017).

### 2.2 Ototoxicity assessment

For case-control assignment in childhood patient cohorts, the SIOP (International Society of Pediatric Oncology) Boston ototoxicity scale was used (Supplementary Table S1). This scale is based on sensorineural hearing thresholds in dB hearing level (Brock et al., 2012). Sensorineural hearing loss was established by examining unaided audiograms showing bone conduction, or air conduction with a normal tympanogram to rule out a conductive hearing loss component. From all patient’s available audiograms during chemotherapy and follow-up, the worst audiogram and the worst ear were scored. Audiograms were scored by trained audiologists or experienced clinicians. For young children (age <4 years), results of otoacoustic emission tests were used instead of audiograms. For the primary analysis, patients with grade 0 were considered controls and patients with grade 1 or higher were assigned as cases. In secondary analyses, patients with grade 0 and 1 were considered controls and patients with grade 2–4 were cases.

### 2.3 Genotyping and quality control

Germline DNA was extracted from blood (collected using QIAamp DNA Blood Midi kit, Qiagen, Venlo, Netherlands) and saliva (collected using GeneFiX DNA Saliva Collector GFX-02, Isohelix, United Kingdom). DNA isolation took place using ChemagicStar (Hamilton Robotics, Reno, NV, United States), using Chemagic STAR DNA Saliva 4k Kit, according to the manufacturer protocol. From patients who had passed away before inclusion, germline DNA was isolated from paraffin-embedded tissue samples as described previously (Hagleitner et al., 2011). All samples were genotyped using genotyping arrays with genome-wide coverage. Samples from the GO-CAT cohort were genotyped using the Illumina Infinium Global Screening Array-24 version 2.0 and version 3.0, performed by Human Genomics Facility at Erasmus MC, Rotterdam, Netherlands. Samples from the United Kingdom MAGIC study cohort were genotyped using the Illumina Infinium OmniExpressExome-8 version 1.4, performed by Illumina Cambridge Ltd., Cambridge, United Kingdom.

Quality control (QC) of genotyped data started with exclusion of samples with individual call rates below 90%. At the marker level, genetic variants were removed if they showed a call rate below 98%, when minor allele frequency (MAF) was below .5% or when they deviated from Hardy Weinberg equilibrium (HWE), with a *p*-value below 1 × 10^−6^. To retain a homogeneous cohort, principal component analysis was performed, using a PCA cut-off of 01 in GO-CAT, and 2 standard deviations of two principal components in United Kingdom MAGIC. Samples were excluded if they showed sex discrepancies between genotyping and phenotyping data, and in cases of relatedness between samples within the cohort (proportion inherited by descent (PI-HAT above .2). To increase number of genetic variants and achieve harmonization across different genotyping arrays, genotyping data from all sites were phased and imputed using software Eagle (v2.3.5) and Minimac3, respectively, with the 1,000 Genomes European dataset as a reference panel (Das et al., 2016; Loh et al., 2016). Imputed variants with an info-score below .6 or a MAF below .5% were excluded. All steps in the process of phasing, imputation and QC in the GO-CAT cohort were performed using the Rapid Imputation and Computational Pipeline for Genome-Wide Association Studies (Lam et al., 2020). In the United Kingdom MAGIC study cohort, identical analyses were performed using multiple command line programs (PLINK was used for QC, Eaglev2.4 for phasing (EUR population) and imputation was performed using the Michigan Server using Minimac4 1.5.7 with the 1000G Phase 3 v5 Reference panel). For genomic location, GRCh37/hg19 was used.

### 2.4 Statistical analysis

A *post hoc* power calculation was performed to estimate the power of detecting a statistical association with the number of available subjects, using Quanto (version 1.2.4, Los Angeles, CA). It was shown that a GWAS analysis in a cohort with sample size of 500, a case-control ratio of 1, a log additive inheritance mode and an alpha of 5 × 10^−8^, would result in a power of 75%–97% for a variant with an odds ratio of 2 and a minor allele frequency of .15–.30. Under these conditions, a power of 80% was reached at minor allele frequencies of .16 or higher. For allele frequencies below .15, the power quickly declines, up to 6% for an allele frequency of .05. Predicted power of this study in scenarios with different odds ratios and minor allele frequencies are presented in Supplementary Figure S1.

To test for potential associations between clinical characteristics and platinum-induced ototoxicity, variables were analyzed using Pearson’s Chi-square, Fisher’s Exact, independent samples T or Mann-Whitney U, depending on the type of data and the Gaussian distribution, using SPSS Statistics (version 25.0, IBM Corp.). Clinical variables with a two-sided *p*-value of less than .05 were included as a covariate in further GWAS analyses. In order to investigate the impact of cranial irradiation, cisplatin/carboplatin treatment and differences in phenotype definition (as explained under Section 2.2) on primary results, exploratory secondary GWAS analyses were performed to evaluate differences in outcome, mainly to evaluate the robustness of top hits. In the primary analysis, patients with SIOP grade 0 are defined as controls and patients with SIOP grade 1–4 as cases. Exploratory secondary analyses were performed in subgroup cohorts with only cisplatin treated patients, non-irradiated patients and different case-control designation (SIOP grade 0–1 compared to grade 2–4), which are included in the Supplementary Material. It is hypothesized that cohorts are more homogenous with more stringent inclusion criteria, leading to lower overall variance in outcome that may outweigh the power loss due to the reduction of patient numbers.

The GWAS was performed as a logistic regression analysis, under the assumption of an additive model, using the software PLINK (v2.0, Cambridge, MA) (Chang et al., 2015). Based on each cohorts’ eigenvalues, the first four (GO-CAT) and two (United Kingdom MAGIC) principal components were included as covariates to account for potential population stratification bias. Age at diagnosis, or age at first cisplatin treatment, and concomitant vincristine treatment were included as covariates in the analysis. The *p*-value threshold for genome-wide statistical significance was set to 5 × 10^−8^. The threshold for suggestive significance was *p*-value of 1 × 10^−5^, which served as a threshold for genetic variants that showed such signal to require further investigation in relation to platinum-induced ototoxicity, but did not reach statistical significance. Summary statistics were shared among GO-CAT and the United Kindgom MAGIC study and a random effect meta-analysis was performed using METAL (v2007–2009 Goncalo Abecasis, released on 05-05-2020) (Willer et al., 2010). Effect size estimates and standard errors from the summary statistics were used to perform the meta-analysis (SCHEME STDERR). In addition, heterogeneity across samples (I^2^) was calculated. Meta-analysis results were filtered for variants that were present in both cohorts (leaving results of 7,272,050 variants in the primary analysis) and were subsequently annotated and visualized using FUMA (v1.3.6b, Amsterdam, Netherlands) (Watanabe et al., 2017). FUMA was used to identify risk loci and their lead SNPs with a *p*-value below the suggestive significance threshold of 1 × 10^−5^. In addition, gene-wide and gene-enrichment analyses were performed using software that was integrated in FUMA (MAGMA).

### 2.5 Replication

Variants suggestively associated to platinum-induced ototoxicity in the primary analysis (*p*-value <1 × 10^−5^) were eligible for replication. The recently published GWAS in the PanCareLIFE cohort was also performed in a childhood cancer cohort, specifically, European, cisplatin-treated, non-cranial irradiated patients with an age of diagnosis <19 years. More details on in and exclusion are reported in their publication (Meijer et al., 2021). Assessment of hearing loss was done using the Muenster grading system, with patients scored as grade 0–2a being controls, and patients with hearing loss ≥ grade 2b were cases. Results of suggestively associated variants from the current study were extracted from publicly available summary statistics (Meijer et al., 2021). The PanCareLIFE data of these variants was combined with the GO-CAT and United Kingdom MAGIC study cohort in a meta-analysis using the same conditions described in Section 2.4 *.*

## 3 Results

### 3.1 Patient population

**FIGURE 1:**
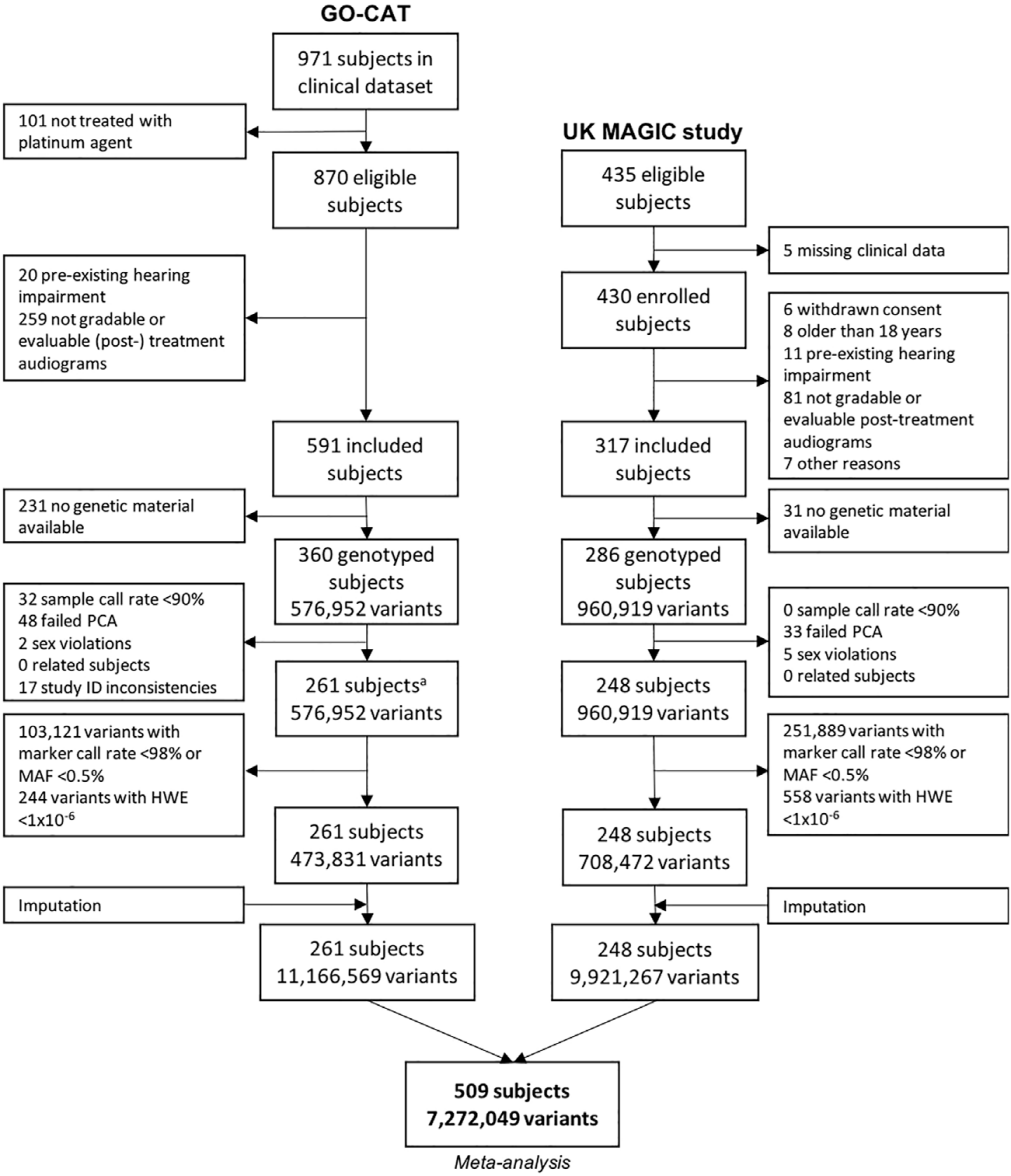
Genetic variant and sample selection flowchart. Process of quality control of clinical and genetic data for GO-CAT cohort and United Kingdom MAGIC study cohort. A quality control of genetic data of GO-CAT cohort was performed in a larger dataset of 848 pediatric oncology subjects, of which a subset of subjects included in this study were extracted after imputation.

The process of patient inclusion is depicted in Figure 1. In summary, of the 971 subjects in the clinical dataset of GO-CAT, a total of 591 subjects met the clinical inclusion criteria, of whom 360 subjects had genetic material available for genotyping. In the UK-MAGIC study cohort, of 435 subjects who were considered eligible, a total of 286 met the inclusion criteria and had genetic material available for genotyping. After quality control of genetic data (Section 3.2), a total of 509 patients remained for GWAS and meta-analysis in the discovery phase of this study, of whom 261 were of the GO-CAT cohort and 248 of the United Kingdom MAGIC study cohort (with originating center of inclusion presented in Supplementary Table S2).

The clinical characteristics of the GO-CAT and United Kingdom MAGIC study cohort are presented in Table 1. Despite variation in diagnoses, the common denominator among these patients was the platinum treatment. The GO-CAT cohort consisted of 136 patients with osteosarcoma (52.1%), 79 with medulloblastoma (30.3%) and 46 with low-grade glioma (17.6%). Of these, 212 patients were primarily treated with cisplatin of which the median cumulative dose was 480 mg/m^2^, ranging from 120 to 900 mg/m^2^ 49 patients were primarily carboplatin treated, and in 21 patients, the primary chemotherapeutic regimen contained both platinum agents, with a median cumulative carboplatin dose of 1,300 mg/m^2^ (range 640–16,047 mg/m^2^). The majority of patients in the United Kingdom MAGIC study cohort were also patients with osteosarcoma (25.5%) or medulloblastoma (25.1%), with the rest being diagnosed with hepatoblastoma (10.9%), neuroblastoma (10.9%), low-grade glioma (5.3%), Hodgkin’s lymphoma (2.0%), ependymoma (4.5%), intracranial germ cell tumor (2.8%), nasopharyngeal carcinoma (2.8%), or even rarer types of childhood cancer. All patients in the United Kingdom MAGIC study cohort received treatment with cisplatin, with a median cumulative dose of 350 mg/m^2^, ranging from 60 to 720 mg/m^2^. A total of 40.2% of patients in the United Kingdom MAGIC study cohort received concomitant carboplatin, mainly for medulloblastoma (16.7%), neuroblastoma (8.9%), hepatoblastoma (4.1%) and ependymoma (4.1%). The use of concomitant vincristine treatment was significantly higher in ototoxicity cases than in controls in the United Kingdom MAGIC cohort (*p* = .001), but not in the GO-CAT cohort (*p* = .092). Concomitant vincristine use was included as a covariate in all GWAS analyses. Males were overrepresented in both cohorts, with 150 males (57.5%) in the GO-CAT cohort and 151 (60.9%) in the United Kingdom MAGIC study cohort, but the proportion of males was not different between cases and controls. The median age ranged from 10.1 to 10.8 years among all groups, except for the ototoxicity cases in the United Kingdom MAGIC study cohort where the median age was 7.9 years. As age was significantly lower in these cases than in controls (*p* = .048), it was included as a covariate in all GWAS analyses.

**TABLE 1:** Demographic data of patients in the discovery cohort of pediatric cancer patients.

		GO-CAT cohort								United Kingdom MAGIC study cohort							
		n*		Controls SIOP grade 0 (n = 124)		Cases SIOP grade 1–4 (n = 137)		*p*-value		n*		Controls SIOP grade 0 (n = 83)		Cases SIOP grade 1–4 (n = 165)		*p*-value	
*Demographics*																	
Male sex (%)		261		73 (53.3%)		77 (62.1%)		.169		248		49 (59.0%)		102 (61.8%)		.681	
Age (years)		261		10.8 (0–38.9)		10.1 (1–41.1)		0.4		247		10.2 (1–18)		7.85 (0–18)		.048	
*Disease and treatment*																	
Diagnosis		261						<.0001		247						<.0001	
	Osteosarcoma			72 (52.6%)		64 (51.6%)						27 (32.5%)		36 (22.0%)			
	Medulloblastoma			27 (19.7%)		52 (41.9%)						3 (3.6%)		59 (36.0%)			
	Other tumors			38 (27.7%)		8 (6.5%)						53 (63.9%)		69 (42.1%)			
Intracranial tumor site (%)		261		65 (47.4%)		60 (48.4%)		.902		247		23 (27.7%)		88 (53.7%)		<.0001	
Received cranial surgery (%)		261		50 (36.5%)		59 (47.6%)		.079		NA		NA		NA		NA	
Received cranial radiotherapy (%)		261		26 (19.0%)		50 (40.3%)		<.0001		248		8 (9.6%)		70 (42.4%)		<.0001	
	Total dose on tumor bed (Gy)	74		54.0 (39.0–60.0)		55.8 (54.0–69.4)		.307		73		55.8 (54–154)		54.4 (32.0–91.8)		<.0001	
Primarily cisplatin treated		261		101 (73.7%)		111 (89.5%)		.0014		248		83 (100%)		165 (100%)		NA	
	Cisplatin cumulative dose (mg/m^2^)	208		480 (120–900)		480 (120–766)		<.0001		248		350 (60–600)		350 (60–720)		.779	
Primarily carboplatin treated		241		41 (33.9%)		29 (24.2%)		.119		0		-		-		-	
	Carboplatin cumulative dose (mg/m^2^)	65		1800 (640–16,047)		1,050 (800–4,400)		.012		0		-		-		-	
*Concomitant ototoxic medication (%)*																	
Vincristine		241		46 (38.0%)		59 (49.2%)		.092		245		29 (35.8%)		97 (52.7%)		.001	
Aminoglycosides		97		6 (11.3%)		9 (20.5%)		.411		NA		NA		NA		NA	
Vancomycin		97		3 (5.7%)		7 (15.9)		.178		NA		NA		NA		NA	
Furosemide		107		9 (15.3%)		2 (4.2%)		.107		NA		NA		NA		NA	
Carboplatin as co-medication (in primarily cisplatin treated patients)		190		3 (3.6%)		16 (15.0%)		.013		246		21 (25.6%)		78 (47.6%)		.001	
*Concomitant otoprotective medication (%)*																	
Amifostine		128		3 (4.2%)		9 (16.1%)		.031		NA		NA		NA		NA	
Sodium Thiosulfate		128		0 (0%)		0 (0%)		-		NA		NA		NA		NA	

Median and range are reported for continuous variables.

^*^ Number of patients with data available for this variable.

NA; not available.

Grade 1–4 platinum-induced ototoxicity occurred in 59.3% (302 cases and 207 controls) of the total discovery cohort. The percentage of cases was higher in the United Kingdom MAGIC cohort (66.5%) than in the GO-CAT cohort (52.5%). In both the GO-CAT and the United Kingdom MAGIC study cohorts, the percentage of patients with cranial irradiation was significantly higher in cases compared to controls (*p* < .0001 in both cohorts). This is related to significant differences in diagnosis, intracranial tumor site, receiving cranial surgery and total dose of radiotherapy received between cases and controls (Table 1). These highly correlated variables are a major effect modifier for ototoxicity and including them as covariates may lead to overcorrection. Therefore, secondary analyses were performed in which patients who received cranial irradiation were excluded to assess how the presence of patients with cranial irradiation influenced the main results. The time interval between platinum treatment and scored audiogram was unavailable for a large proportion of patients, so could therefore not be analyzed.

### 3.2 Genotyping

The process of genotyping, QC and imputation for the GO-CAT cohort was performed in a larger cohort of pediatric cancer patients (848 subjects), of which a subset of subjects was eligible for and included in this study (Figure 1). Genotyping in the UK-MAGIC cohort was performed in 286 subjects. The process of QC for both cohorts is depicted in Figure 1. A total of 81 subjects (being 48 in GO-CAT and 33 in UK-MAGIC) were excluded in PCA due to non-European descent. After QC, imputation and matching clinical and genetic data, a total of 261 subjects and 11,166,569 variants were included from the GO-CAT cohort, and a total of 248 subjects and 9,921,267 variants in the UK-MAGIC study cohort. Combining both cohorts in the meta-analysis resulted in a total of 509 subjects and 7,272,049 genetic variants.

### 3.3 Genome-wide association analyses and meta-analysis

**FIGURE 2:**
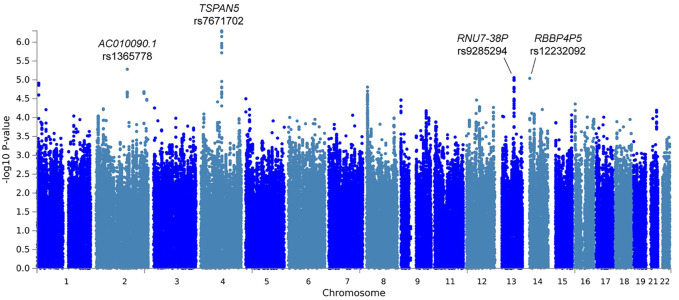
Manhattan plot of the primary GWAS meta-analysis, including patients treated with cisplatin or carboplatin and patients with and without cranial irradiation. In this analysis, patients with SIOP grade 0 are considered controls and patients with SIOP grade ≥1 are cases.

**FIGURE 3:**
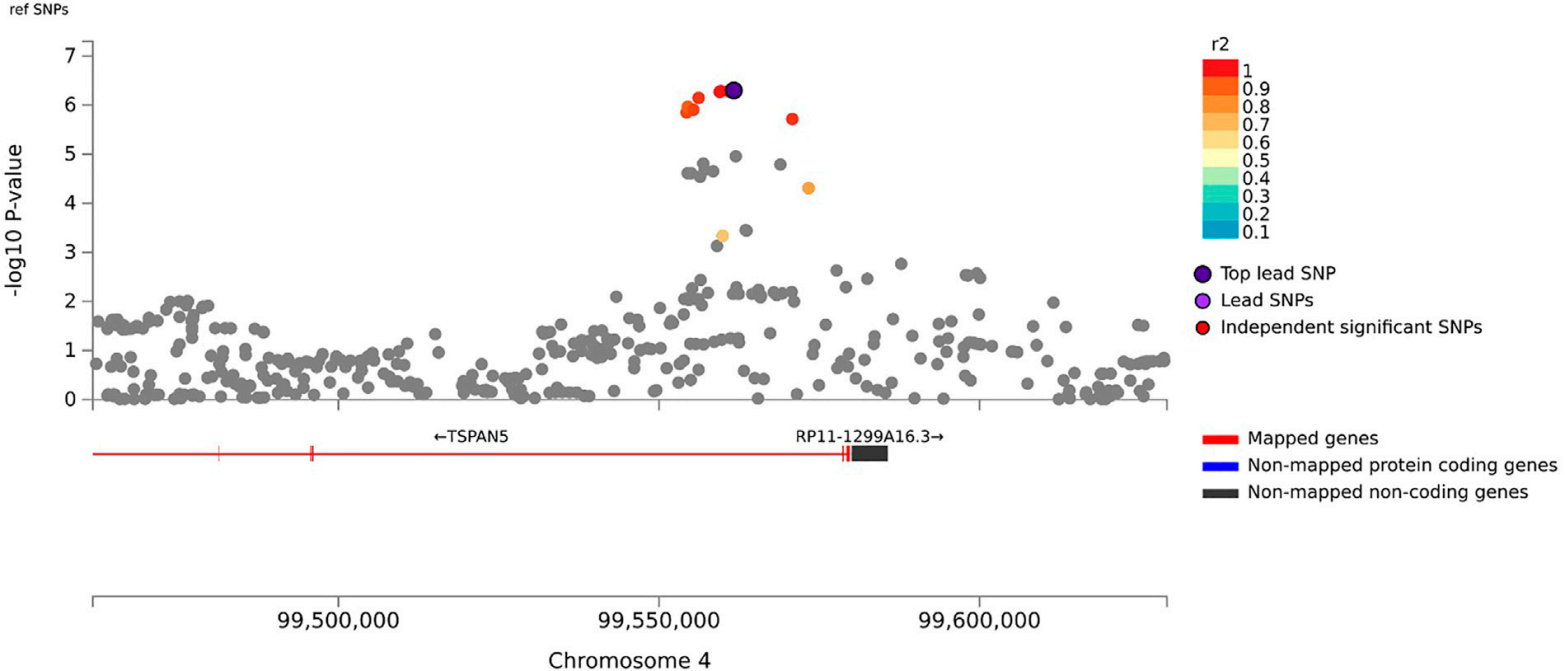
Zoom plot of rs7671702 and its surrounding region on chromosome 4. This variant (purple diamond) is located in the intronic region of the *TSPAN5*. *p*-values on the-log 10 scale are plotted on the left *y*-axis. The right *y*-axis indicates the regional recombination rate (cM/Mb), depicted by the blue line in the plot (where peaks indicate recombination hot spots). The chromosomal position is plotted along the *x*-axis along with the genes located in that region. Variants that are in linkage disequilibrium (r2) with this variant are indicated by colored dots (where different colors represent the level of linkage, as stated in the legend in the upper right corner).

In the primary meta-analysis, four variants were suggestively associated with platinum-induced ototoxicity (*p*-value < 1 × 10^−5^, Table 2 and Figure 2). Of these, rs7671702 in *TSPAN5* on chromosome four showed the lowest *p*-value (Figure 2). The T-allele of this variant was shown to increase the risk of ototoxicity (OR = 2.0, 95% CI = 1.5–2.7) in childhood cancer patients primarily treated with cisplatin or carboplatin (*p* = 5.0 × 10^−7^) (Table 2). Figure 3 shows that the *TSPAN5* locus was intronic, where multiple other genetic variants, which are in high linkage disequilibrium, also showed an association. The other variants that were suggestively associated with platinum-induced ototoxicity were located in *RBBP4P5, AC010090.1* and *RNU6-38P*. The quantile-quantile plot of this analysis (Supplementary Figure S2) shows that the GWAS meta-analysis was underpowered.

**TABLE 2:** Results of the genetic variants that are suggestively associated to platinum-induced hearing loss in the primary GWAS meta-analysis. The GO-CAT and United Kingdom MAGIC study cohorts form together the discovery cohort. Results are specified per cohort and the combined results are determined with a meta-analysis.

	GWAS GO-CAT consortium n = 261		GWAS United Kingdom MAGIC study cohort n = 245		Meta-analysis (GO-CAT + United Kingdom MAGIC)			Replication PanCareLIFE cohort n = 390		Meta-analysis (GO-CAT + United Kingdom MAGIC + PanCareLIFE)		
	OR	*p*-value	OR	*p*-value	OR	*p*-value	I^2^	OR	*p*-value	OR	*p*-value	I^2^
	(95% CI)		(95% CI)		(95% CI)		P_het_	(95% CI)		(95% CI)		P_het_
*TSPAN5* rs7671702 (A1 = T)	1.8	1.8 × 10^−3^	2.3	2.4 × 10^−5^	2.0	5.0 × 10^−7^	0	1.2	.24	1.6	8.9 × 10^−6^	73.6
	(1.3–2.7)		(1.6–3.4)		(1.5–2.7)		.411	(.9–1.6)		(1.3–1.9)		.023
*RBBP4P5* rs12232092 (A1 = A)	2.5	1.2 × 10^−3^	2.0	2.1 × 10^−3^	2.2	9.1 × 10^−6^	0	1.2	.28	1.7	7.1 × 10^−5^	63.4
	(1.4–4.4)		(1.3–3.2)		(1.6–3.1)		.545	(.9–1.8)		(1.3–2.2)		.065
*AC010090.1* rs1365778 (A1 = A)	2.4	6.4 × 10^−4^	2.0	2.4 × 10^−3^	2.2	5.2 × 10^−6^	0	1.2	.33	1.6	8.9 × 10^−5^	69.5
	(1.5–3.9)		(1.3–3.1)		(1.6–3.1)		.644	(.8–1.7)		(1.3–2.0)		.038
*RNU6-38P* rs9285294 (A1 = T)	0.5	2.1 × 10^−4^	0.6	1.0 × 10^−2^	0.5	8.8 × 10^−6^	0	1.0	.99	0.7	1.1 × 10^−3^	79.4
	(.3–.7)		(.4–.9)		(.4–.7)		.441	(.7–1.4)		(.6–.9)		.008

A1, effect allele; OR, odds ratio; 95%CI, 95% confidence interval; I^2^. Heterogeneity value.

**FIGURE 4:**
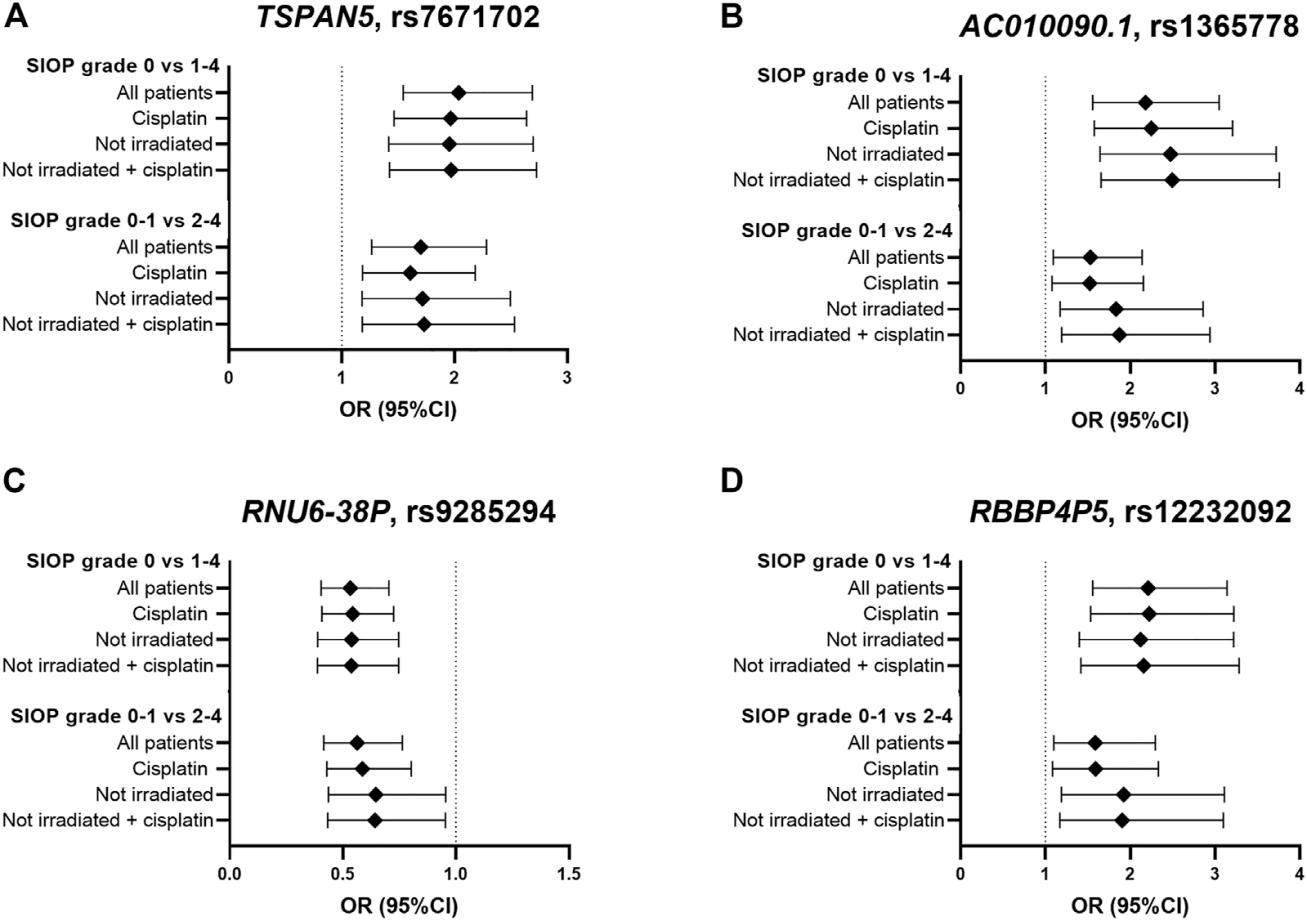
Results in primary and secondary analyses of four variants **(A–D)** that were suggestively associated with platinum-induced ototoxicity in the primary GWAS meta-analysis. The top bar represents the primary analysis (SIOP grade 0 vs 1–4, “all patients”). In the “cisplatin” subgroup, patients treated with carboplatin were excluded and in the ‘not irradiated’ subgroup, patients that received cranial irradiation were excluded.

In total, eight genome-wide association analyses were performed according to the same protocols, with varying inclusion criteria and phenotype definitions. These analyses consisted of subgroups with only cisplatin treated patients and non-irradiated patients, and using a different case-control designation (SIOP grade 0–1 compared to grade 2–4). The four strongest associated variants from the primary analysis were investigated in the results of the secondary analyses, which are shown in Figure 4 and Supplementary Table S3. In the analysis with only cisplatin-treated patients, the variants in *TSPAN5* and *AC010090.1* remain the strongest associated variants and surpass the *p*-value threshold of 10^−5^. When restricting the analysis to patients without cranial irradiation, excluding 154 irradiated patients, power of the analysis decreased, resulting in lower precision of estimates and larger *p*-values. Despite that, the direction of effect of the four variants was consistent across all secondary analyses. The variants surpassing the *p*-value threshold of 10^−5^ from every GWAS are depicted in Supplementary Table S4.

In the primary gene-wide and gene-enrichment analysis, no statistically significant associations were found after Bonferroni or FDR multiple testing correction (data not shown). Despite the non-significant results, the *GBP1* gene in chromosome 1 is in the top 5 in all gene-wide analyses, suggesting a consistency of this finding despite the primarily exact inclusion criteria of the patient cohorts.

### 3.4 Replication

Four variants were eligible for replication in the PanCareLIFE cohort. This cohort consisted of 390 cisplatin-treated, non-cranially irradiated childhood cancer patients. Forty-three percent (n = 168) of these patients suffered from cisplatin-induced ototoxicity (Muenster ≥ grade 2b), with details on patient demographics provided in their publication (Meijer et al., 2021). None of the associations were statistically significant in the replication cohort, but, with exception of rs9285294 with OR of 1, concordance in the direction of effect was observed (Table 2). In a meta-analysis of all pediatric cancer cohorts (GO-CAT, United Kingdom MAGIC and PanCareLIFE), the effect of the *TSPAN5* variant remained suggestively significant (OR = 1.6 (95% CI = 1.3–1.9), *p* = 8.9 × 10^−6^, I^2^ = 73.6%).

## 4 Discussion

This study did not identify genetic variants statistically significantly associated to platinum-induced ototoxicity. A genetic variant in the *TSPAN5* gene with platinum-induced ototoxicity in pediatric cancer patients emerged as main candidate of interest. In addition, this study identified variants *in RBBP4P5, AC010090.1* and *RNU6-38P* that approached statistical significance. These findings combined with previously published studies show that germline genetic variation may play a role in the development of ototoxicity after platinum treatment.

Genetic variant rs7671702 is located in the first intron of the *TSPAN*5 gene. This variant has previously been found to be an expression quantitative trait locus (eQTL) for *TSPAN5* in skeletal muscle and tibial nerves, meaning that the variant is associated with expression of *TSPAN5* (GTEx Consortium, 2020). *TSPAN5* codes for tetraspanin 5, which is a ubiquitously expressed protein that is responsible for the translocation of ADAM10 to the cell membrane (Matthews et al., 2017). ADAM10 is involved in cisplatin-induced renal toxicity through cleaving C-X-C ligand 16 (CXCL16) into its soluble form, causing recruitment of T Cells and subsequent inflammation-mediated apoptosis (Aboyoussef et al., 2021). Interestingly, it has been showed that the low molecular weight heparin enoxaparin relieves platinum-induced nephrotoxicity *in vitro* (Aboyoussef et al., 2021). Also, regulation of ADAM10 by PAX2 or miR-320a influences cisplatin sensitivity of melanoma cells and gastric cancer cells, respectively (Lee et al., 2011; Ge et al., 2017). This indicates that ADAM10 translocation by TSPAN5 may also affect sensitivity to platinum compounds. In addition, ADAM10 regulates sensory regeneration in avian vestibular organs (Warchol et al., 2017). In previous gene-wide analyses, it was shown that *TSPAN5* was associated to tinnitus (uncorrected *p* = .00187 (Watanabe et al., 2019)) and that there is an association of *ADAM10* with cisplatin-induced ototoxicity (uncorrected *p* = .0466 (Wheeler et al., 2017)). However, in the gene-wide analysis of this study, *TSPAN5* showed a *p*-value of 8.65 × 10^−4^, but *ADAM10* did not show this trend towards (*p* = .78) in the primary analysis. These results indicate that there may be a role for *TSPAN5* in platinum-induced ototoxicity, but that mechanism behind this role is yet to be determined.

Despite the fact that there could be a mechanistic explanation for the association between *TSPAN5* variant and platinum-induced ototoxicity, this association was not found in the study in the PanCareLIFE cohort published by Meijer et al. (2021). Vice versa, the association between *TCERG1L* rs893507 and cisplatin-induced ototoxicity, discovered in the PanCareLIFE cohort, was not replicated in primary nor secondary analyses of this study (Supplementary Table S5). It should be noted that this variant was not among the analyzed variants in the United Kingdom MAGIC cohort, and could therefore only be investigated in the GO-CAT cohort. When comparing this study with the PanCareLIFE study, the patient cohorts were relatively comparable, but the definition of ototoxicity was different. In the current study’s primary analysis, patients with SIOP grade ≥1 were considered as cases (>20 dB hearing loss at > 4 kHz) while in the PanCareLIFE study, hearing loss was considered deleterious at Muenster level 2b (>40 dB hearing loss at ≥ 4 kHz). The criteria from the PanCareLIFE study are therefore more stringent compared to this study’s criteria. In secondary analyses the current study, SIOP grade ≥2 cases (>20 dB hearing loss at ≥ 4 kHz) would be more comparable to the PanCareLIFE study. Secondary analyses were also more comparable to the PanCareLIFE study in terms of inclusion criteria, because PanCareLIFE only included cisplatin-treated, non-irradiated patients. Different thresholds in the case-control designation can have a large effect on the results of a genetic association study, as previously described in a study focusing on cisplatin induced nephrotoxicity (Zazuli et al., 2019). They used four grading tools to represent acute kidney injury, including CTCAE grading, adjusted CTCAE grading, serum creatinine and estimated glomerular filtration rate. It was found that these different case designations lead to variability in risk ascertainment of the phenotype. This effect was also illustrated by differences in results in the analyses with cases defined as SIOP grade ≥1 compared to cases defined as SIOP grade ≥2, respectively (Figure 4). Altogether, this highlights the importance of homogenous clinically relevant outcome definitions.

In the primary association analysis of this study, both cis- and carboplatin treated patients were analyzed together. Despite known differences in ototoxic potency of these agents, cisplatin being more ototoxic than carboplatin, the mechanism of ototoxicity is likely to be through the same biological pathways, with death of sensory hair cells in the cochlea being the endpoint (Brock et al., 2012). Since the aim of this genetic association study was to identify potential pathways *via* statistical methods in order to increase knowledge about interindividual differences, patients treated with either or both agents were included in the primary analyses largely to enhance power by increasing the cohort size. However, exclusion of patients treated primarily with carboplatin (n = 50), although reducing the variance in the data, resulted in decreased power.

A few patients (n = 12) were treated with the otoprotective agents amifostine, however these patients were not excluded from the analysis as the number of patients is very small and no differences in amifostine use between cases and controls were observed. Therefore it is highly likely that this will not have an effect on the results. Besides amifostine was given as part of a research study and the patients showed no signs of protection against hearing loss. Also the most recent systematic review studying the otoprotective effect of amifostine in children treated with platinum based therapy, including two randomized controlled trials and one controlled clinical trial, concluded that based on the studies performed to date, there is no evidence that amifostine is indeed otoprotective (van As et al., 2019).

Despite inclusion of a relatively large number of patients in the field of pharmacogenetics in pediatric oncology, made possible through collaboration with multiple research groups, no genome-wide significant associations were found and the results could not be confirmed in the PanCareLIFE cohort. Fifteen genetic variants that were previously associated with platinum-induced ototoxicity were not significantly associated with platinum-induced ototoxicity in this study (Supplementary Table S5). The lack of replication could be due to heterogeneity between studies, e.g., differences in outcome definitions and methods of analysis, false-positive findings in the discovery studies, or insufficient power. Poor reproducibility remains an issue in genetic association studies, including in platinum-induced ototoxicity for these reasons (Drogemoller et al., 2019). In the quest for meaningful associations with an impact on patient care, homogenous and powerful analyses with larger patient cohorts are necessary.

This study once again emphasizes the importance of standardized outcome definitions, homogeneous analyses and collaboration among research groups to optimize power in GWAS studies. Statistical power is a critical issue which further suggests that the effect size for this phenotype will be relatively modest (as opposed to very large effect sizes observed with other pharmacogenomic phenotypes such as drug hypersensitivity reactions), and it is possible there may be multiple loci, all with low to moderate effect sizes contributing to susceptibility. Therefore, the Genetics of Childhood Cancer Treatment (GO-CAT) consortium will continue to invest in collaborations to perform larger analyses in the future. This exploratory study adds to the existing knowledge regarding involvement of genetic variants in heterogenicity of platinum-induced ototoxicity. This may contribute to an improved understanding of the mechanism(s) behind platinum-induced ototoxicity.

## Data Availability

The datasets presented in this study can be found in online repositories. The names of the repository/repositories and accession number(s) can be found in the article/supplementary material.
