# Supplementary material for: Genome-wide analyses of platinum-induced ototoxicity in childhood cancer patients: Results of GO-CAT and United Kingdom MAGIC consortia
Source: Front Pharmacol. 2023 Jan 9;13:980309. doi: 10.3389/fphar.2022.980309 (PMC9870026; doi:10.3389/fphar.2022.980309)
Supplement: Supplementary file 1 [file DataSheet1.PDF]

Supplementary Material

***Table S1*. Hearing levels that are designated to toxicity grades** according to SIOP Boston ototoxicity scale for pediatric patients (2).

	**SIOP Boston**
**Grade 0**	≤ 20 dB all
**Grade 1**	>20 dB at >4 kHz
**Grade 2**	>20 dB at ≥4 kHz
**Grade 3**	>20 dB at 2 or 3 kHz
**Grade 4**	>40 dB at ≥2 kHz

***Table S2*. Hospital of inclusion** of the patients in the discovery and replication cohorts.

	**Controls**	**Cases**
**Genetics of Childhood Cancer Treatment (GO-CAT) consortium**		
Radboudumc Nijmegen, The Netherlands	45	27
Leiden UMC, The Netherlands	4	12
AMC, Amsterdam, The Netherlands	6	11
UMC Groningen, The Netherlands	4	12
The Children's Hospital at Westmead, Australia	27	16
Fondazione IRCCS Istituto Nazionale Tumori Milan, Italy	37	31
**UK MAGIC study cohort**		
Alder Hey Children’s Hospital, Liverpool, England	16	30
Great Ormond Street Hospital NHS Trust, London, UK	5	13
Leeds General Infirmary, Leeds, UK	21	43
Leicester Royal Infirmary NHS Trust, Leicester, UK	1	3
Nottingham University Hospitals NHS Trust, Nottingham, UK	28	50
Newcastle Hospitals NHS Trust, Newcastle, UK	4	12
Royal Manchester Children’s Hospital, Manchester, UK	8	11
York Hill Hospital, Glasgow, UK	0	3
**PanCareLIFE**		
Available at: Meijer AJM, Diepstraten FA, Langer T, Broer L, Domingo IK, Clemens E, et al. TCERG1L allelic variation is associated with cisplatin-induced hearing loss in childhood cancer, a PanCareLIFE study. NPJ Precis Oncol. 2021;5(1):64.		

***Figure S1*. Results of power calculations for platinum-induced ototoxicity in 509 patients**

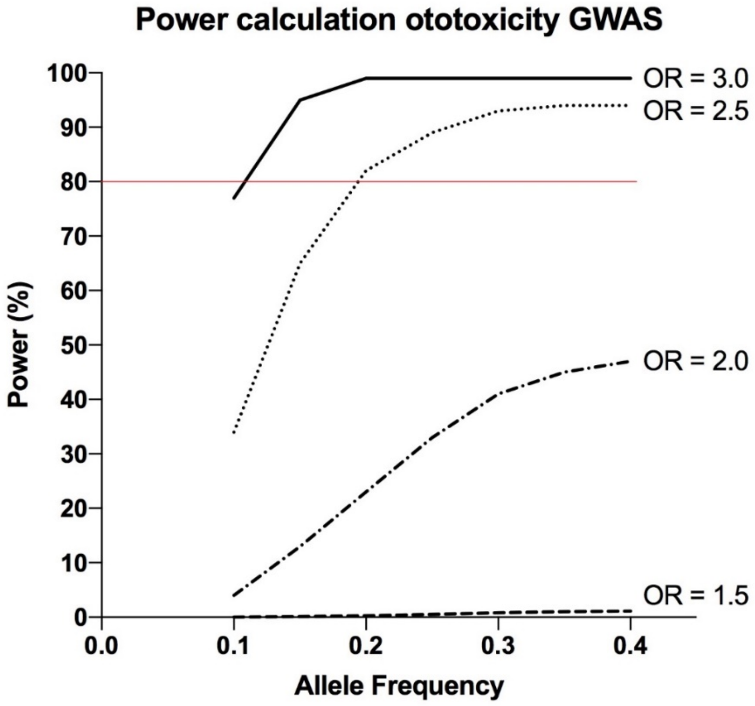

***Figure S2*. QQ-plot of the primary GWAS meta-analysis** to identify genetic variants associated to platinum-induced hearing loss.

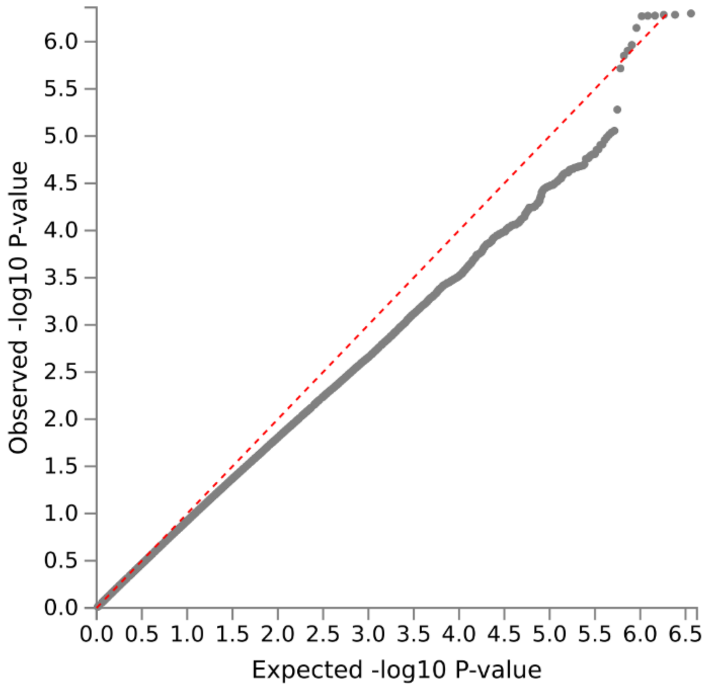

***Table S3*. Four variants were suggestively associated with platinum-induced ototoxicity in primary analysis** and this table shows the results of these associations in the secondary analyses. Selection criteria of patients in primary (1A) and secondary analyses (1B-4B) are specified in this table. In the 'cisplatin' subgroup, patients treated with carboplatin were excluded and in the 'not irradiated' subgroup, patients that received cranial irradiation were excluded.

***Gene*, rsID**	**#**	**Inclusion criteria**	**Control**<br>SIOP grade	**Case**<br>SIOP grade	**N**	**OR (95% CI)**	**Uncorrected *p*-value**
***TSPAN5*, rs7671702**	1A	All patients	0	1-4	506	2.21 (1.56 - 3.14)	9.112E-06
	1B	Cisplatin	0	1-4	456	2.22 (1.53 - 3.22)	2.491E-05
	2A	Not irradiated	0	1-4	352	2.12 (1.4 - 3.22)	0.0004074
	2B	Not irradiated + Cisplatin	0	1-4	331	2.16 (1.42 - 3.28)	0.0003275
	3A	All patients	0-1	2-4	506	1.59 (1.1 - 2.29)	0.01363
	3B	Cisplatin	0-1	2-4	456	1.59 (1.08 - 2.33)	0.01757
	4A	Not irradiated	0-1	2-4	352	1.92 (1.19 - 3.11)	0.007856
	4B	Not irradiated + Cisplatin	0-1	2-4	331	1.9 (1.17 - 3.1)	0.009534
***AC010090.1*, rs1365778**	1A	All patients	0	1-4	506	2.18 (1.56 - 3.05)	5.235E-06
	1B	Cisplatin	0	1-4	456	2.25 (1.58 - 3.2)	7.569E-06
	2A	Not irradiated	0	1-4	352	2.47 (1.64 - 3.72)	1.363E-05
	2B	Not irradiated + Cisplatin	0	1-4	331	2.49 (1.66 - 3.76)	1.239E-05
	3A	All patients	0-1	2-4	506	1.53 (1.09 - 2.14)	0.01302
	3B	Cisplatin	0-1	2-4	456	1.52 (1.08 - 2.15)	0.0172
	4A	Not irradiated	0-1	2-4	352	1.83 (1.17 - 2.86)	0.007635
	4B	Not irradiated + Cisplatin	0-1	2-4	331	1.87 (1.19 - 2.94)	0.006409
***RNU6-38P*, rs9285294**	1A	All patients	0	1-4	506	2.04 (1.54 - 2.69)	5.013E-07
	1B	Cisplatin	0	1-4	456	1.96 (1.46 - 2.64)	7.081E-06
	2A	Not irradiated	0	1-4	352	1.95 (1.42 - 2.7)	4.562E-05
	2B	Not irradiated + Cisplatin	0	1-4	331	1.97 (1.42 - 2.73)	4.361E-05
	3A	All patients	0-1	2-4	506	1.7 (1.27 - 2.28)	0.0004241
	3B	Cisplatin	0-1	2-4	456	1.61 (1.18 - 2.18)	0.002354

	4A	Not irradiated	0-1	2-4	352	1.72 (1.18 - 2.49)	0.00468
	4B	Not irradiated + Cisplatin	0-1	2-4	331	1.73 (1.18 - 2.53)	0.004764
***RBBP4P5*, rs12232092**	1A	All patients	0	1-4	506	0.53 (0.4 - 0.7)	8.758E-06
	1B	Cisplatin	0	1-4	456	0.54 (0.41 - 0.72)	3.358E-05
	2A	Not irradiated	0	1-4	352	0.54 (0.39 - 0.75)	0.0002025
	2B	Not irradiated + Cisplatin	0	1-4	331	0.54 (0.39 - 0.75)	0.0002073
	3A	All patients	0-1	2-4	506	0.56 (0.41 - 0.76)	0.0002158
	3B	Cisplatin	0-1	2-4	456	0.59 (0.43 - 0.8)	0.0008312
	4A	Not irradiated	0-1	2-4	352	0.65 (0.44 - 0.95)	0.02829
	4B	Not irradiated + Cisplatin	0-1	2-4	331	0.64 (0.43 - 0.95)	0.02796

Table S4. **Results of the suggestive associations ($p<1\times10^{-5}$) of primary (1A) and secondary (1B-4B) GWAS meta-analyses in the discovery cohort**

#*	rsID	chr	Position	Nearest Gene	Distance	Non effect allele	Effect allele	MAF	Effect direction[1]	N	OR	95 CI (lower)	95 CI (upper)	Uncorrected *p*-value
**1A**	rs7671702	4	99561695	***TSPAN5***	0	C	T	0.491	++	506	2.04	1.76	2.32	**5.01E-07**
	rs1365778	2	145305277	***AC010090.1***	18724	G	A	0.327	++	506	2.18	1.84	2.51	**5.24E-06**
	rs9285294	13	75672116	***RNU6-38P***	11607	C	T	0.453	--	506	0.53	0.26	0.81	**8.76E-06**
	rs12232092	14	22028781	***RBBP4P5***	3168	G	A	0.275	++	506	2.21	1.86	2.56	**9.11E-06**
**1B**	rs7671702	4	99561695	***TSPAN5***	0	C	T	0.491	++	456	1.96	1.67	2.26	**7.08E-06**
	rs1365778	2	145305277	***AC010090.1***	18724	G	A	0.327	++	456	2.25	1.89	2.60	**7.57E-06**
**2B**	rs11173964	12	61795352	***RP11-471N19.1***	168633	C	T	0.392	++	331	2.20	1.86	2.54	**5.16E-06**
**3A**	rs2800881	1	112133910	***RAP1A***	0	C	T	0.358	++	506	2.07	1.75	2.39	**8.68E-06**
**3B**	rs2800881	1	112133910	***RAP1A***	0	C	T	0.358	++	456	2.14	1.81	2.48	**6.75E-06**
	rs11058553	12	126635113	***RP1-116K23.1***	17667	G	A	0.255	--	456	0.43	0.05	0.80	**9.48E-06**
**4A**	rs9973644	2	21648283	***AC067959.1:AC011752.1***	0	C	T	0.320	--	352	0.38	-0.03	0.80	**5.45E-06**
**4B**	rs13403903	2	21609157	***AC067959.1:AC011752.1***	0	C	T	0.342	++	331	2.58	2.16	3.00	**9.57E-06**

MAF, minor allele frequency; OR, odds ratio; CI, confidence interval.

* case-control designation and inclusion criteria of each analysis are specified in *Table S3*.

[1] Effect direction represents the effect direction in the GO-CAT cohort and the UK MAGIC cohort, respectively, where '+' means that the effect allele is linked to increased risk to develop the phenotype (OR > 1) and '–' to decreased risk (OR < 1).

***Table S5*. Results of genetic variants that were associated to platinum-induced ototoxicity in previous studies, in the GWAS meta-analysis of this study**

#*	Gene	SNP	Allele 1	Allele 2	Effect direction[1]	OR (95CI)	Uncorrected *p*-value
1B	*ABCC3*	rs1051640	a	g	--	0.87 (0.6 - 1.27)	0.476
1A	*ABCC3*	rs1051640	a	g	+-	0.91 (0.64 - 1.3)	0.603
4B	*ABCC3*	rs1051640	a	g	++	1.12 (0.67 - 1.88)	0.666
4A	*ABCC3*	rs1051640	a	g	-+	1.1 (0.66 - 1.82)	0.714
2A	*ABCC3*	rs1051640	a	g	-+	0.95 (0.62 - 1.45)	0.802
3B	*ABCC3*	rs1051640	a	g	-+	0.95 (0.64 - 1.42)	0.811
2B	*ABCC3*	rs1051640	a	g	-+	0.96 (0.62 - 1.48)	0.858
3A	*ABCC3*	rs1051640	a	g	-+	1.03 (0.7 - 1.51)	0.89
3B	*ACYP2*	rs1872328	a	g	--	0.58 (0.19 - 1.78)	0.339
3A	*ACYP2*	rs1872328	a	g	--	0.63 (0.23 - 1.72)	0.364
4A	*ACYP2*	rs1872328	a	g	--	0.77 (0.23 - 2.62)	0.68
4B	*ACYP2*	rs1872328	a	g	+-	0.82 (0.24 - 2.79)	0.748
1B	*ACYP2*	rs1872328	a	g	++	1.17 (0.44 - 3.08)	0.757
2B	*ACYP2*	rs1872328	a	g	++	1.1 (0.38 - 3.13)	0.865
2A	*ACYP2*	rs1872328	a	g	++	1.07 (0.38 - 3.01)	0.903
1A	*ACYP2*	rs1872328	a	g	-+	1 (0.42 - 2.37)	0.999
3A	*GSTP1*	rs1695	a	g	+-	0.77 (0.56 - 1.06)	0.112
3B	*GSTP1*	rs1695	a	g	+-	0.79 (0.57 - 1.1)	0.16
2B	*GSTP1*	rs1695	a	g	+-	0.8 (0.56 - 1.14)	0.214
1A	*GSTP1*	rs1695	a	g	+-	0.84 (0.62 - 1.13)	0.245
1B	*GSTP1*	rs1695	a	g	+-	0.83 (0.61 - 1.14)	0.251
4B	*GSTP1*	rs1695	a	g	+-	0.8 (0.53 - 1.21)	0.294
2A	*GSTP1*	rs1695	a	g	+-	0.83 (0.58 - 1.18)	0.302
4A	*GSTP1*	rs1695	a	g	+-	0.82 (0.54 - 1.23)	0.336
1B	*LRP2*	rs2075252	t	c	++	1.16 (0.82 - 1.64)	0.402
1A	*LRP2*	rs2075252	t	c	++	1.06 (0.76 - 1.47)	0.726
2A	*LRP2*	rs2075252	t	c	+-	0.96 (0.66 - 1.39)	0.817
3B	*LRP2*	rs2075252	t	c	-+	1.04 (0.72 - 1.52)	0.823
3A	*LRP2*	rs2075252	t	c	-+	0.97 (0.67 - 1.4)	0.87
4A	*LRP2*	rs2075252	t	c	+-	0.98 (0.63 - 1.54)	0.937
4B	*LRP2*	rs2075252	t	c	+-	1.02 (0.65 - 1.59)	0.948

2B	*LRP2*	rs2075252	t	c	+-	0.99 (0.68 - 1.45)	0.976
**2B**	***NFE2L2***	**rs6721961**	**t**	**g**	**--**	**0.57 (0.32 - 1)**	**0.049**
2A	*NFE2L2*	rs6721961	t	g	--	0.59 (0.34 - 1.03)	0.063
1B	*NFE2L2*	rs6721961	t	g	--	0.69 (0.43 - 1.08)	0.106
1A	*NFE2L2*	rs6721961	t	g	--	0.73 (0.48 - 1.13)	0.162
4B	*NFE2L2*	rs6721961	t	g	--	0.68 (0.35 - 1.33)	0.261
4A	*NFE2L2*	rs6721961	t	g	--	0.7 (0.37 - 1.35)	0.287
3A	*NFE2L2*	rs6721961	t	g	--	0.89 (0.57 - 1.4)	0.624
3B	*NFE2L2*	rs6721961	t	g	+-	0.93 (0.59 - 1.47)	0.758
4A	*OTOS*	rs2291767	t	c	-+	2.05 (0.7 - 6.06)	0.193
4B	*OTOS*	rs2291767	t	c	-+	1.97 (0.67 - 5.84)	0.22
2A	*OTOS*	rs2291767	t	c	++	1.63 (0.61 - 4.4)	0.332
2B	*OTOS*	rs2291767	t	c	++	1.63 (0.6 - 4.41)	0.337
1B	*OTOS*	rs2291767	t	c	++	1.45 (0.58 - 3.65)	0.431
1A	*OTOS*	rs2291767	t	c	++	1.34 (0.58 - 3.1)	0.49
3A	*OTOS*	rs2291767	t	c	-+	1.07 (0.46 - 2.5)	0.88
3B	*OTOS*	rs2291767	t	c	-+	1.04 (0.42 - 2.6)	0.935
3A	*SLC16A5*	rs4788863	t	c	++	1.32 (0.95 - 1.83)	0.096
4B	*SLC16A5*	rs4788863	t	c	++	1.42 (0.92 - 2.2)	0.115
4A	*SLC16A5*	rs4788863	t	c	++	1.4 (0.91 - 2.15)	0.131
3B	*SLC16A5*	rs4788863	t	c	++	1.22 (0.87 - 1.71)	0.26
1A	*SLC16A5*	rs4788863	t	c	+-	1.17 (0.86 - 1.6)	0.319
2B	*SLC16A5*	rs4788863	t	c	+-	1.15 (0.8 - 1.66)	0.445
1B	*SLC16A5*	rs4788863	t	c	+-	1.12 (0.81 - 1.55)	0.482
2A	*SLC16A5*	rs4788863	t	c	+-	1.13 (0.79 - 1.63)	0.494
4A	*SLC22A2*	rs316019	a	c	++	1.14 (0.61 - 2.12)	0.69
2A	*SLC22A2*	rs316019	a	c	+-	1.05 (0.62 - 1.8)	0.849
3A	*SLC22A2*	rs316019	a	c	--	0.96 (0.59 - 1.54)	0.853
3B	*SLC22A2*	rs316019	a	c	--	0.95 (0.58 - 1.57)	0.855
4B	*SLC22A2*	rs316019	a	c	++	1.05 (0.55 - 2.01)	0.874
1B	*SLC22A2*	rs316019	a	c	+-	1.02 (0.63 - 1.63)	0.948
1A	*SLC22A2*	rs316019	a	c	+-	1.01 (0.65 - 1.58)	0.952
2B	*SLC22A2*	rs316019	a	c	+-	0.99 (0.58 - 1.71)	0.981
2A	*SOD2*	rs4880	a	g	--	0.76 (0.55 - 1.07)	0.114
2B	*SOD2*	rs4880	a	g	--	0.77 (0.55 - 1.08)	0.129
1A	*SOD2*	rs4880	a	g	--	0.83 (0.62 - 1.1)	0.199

1B	*SOD2*	rs4880	a	g	--	0.85 (0.63 - 1.14)	0.268
3B	*SOD2*	rs4880	a	g	--	0.85 (0.63 - 1.15)	0.296
3A	*SOD2*	rs4880	a	g	--	0.87 (0.64 - 1.17)	0.344
4B	*SOD2*	rs4880	a	g	+-	0.85 (0.57 - 1.25)	0.403
4A	*SOD2*	rs4880	a	g	+-	0.86 (0.58 - 1.27)	0.445
1B	*TCERG1L*	rs893507	t	c	+?	1.28 (0.72 - 2.31)	0.402
1A	*TCERG1L*	rs893507	t	c	+?	1.15 (0.68 - 1.93)	0.607
2A	*TCERG1L*	rs893507	t	c	+?	1.14 (0.61 - 2.12)	0.675
2B	*TCERG1L*	rs893507	t	c	+?	1.14 (0.61 - 2.13)	0.675
3A	*TCERG1L*	rs893507	t	c	-?	0.88 (0.47 - 1.65)	0.7
4B	*TCERG1L*	rs893507	t	c	-?	0.88 (0.39 - 1.96)	0.747
4A	*TCERG1L*	rs893507	t	c	-?	0.92 (0.41 - 2.04)	0.833
3B	*TCERG1L*	rs893507	t	c	+?	1.06 (0.52 - 2.15)	0.872
4A	*TPMT*	rs1142345	t	c	++	2.23 (0.92 - 5.39)	0.076
4B	*TPMT*	rs1142345	t	c	++	2.22 (0.92 - 5.38)	0.076
3B	*TPMT*	rs1142345	t	c	++	1.54 (0.75 - 3.14)	0.24
3A	*TPMT*	rs1142345	t	c	++	1.52 (0.75 - 3.09)	0.245
1B	*TPMT*	rs1142345	t	c	-+	1.1 (0.56 - 2.18)	0.783
2B	*TPMT*	rs1142345	t	c	-+	0.95 (0.45 - 2.01)	0.887
2A	*TPMT*	rs1142345	t	c	-+	0.95 (0.45 - 2)	0.889
1A	*TPMT*	rs1142345	t	c	-+	1.04 (0.54 - 2)	0.907
4A	*TPMT*	rs12201199	a	t	++	1.8 (0.84 - 3.88)	0.132
4B	*TPMT*	rs12201199	a	t	++	1.79 (0.83 - 3.86)	0.136
3A	*TPMT*	rs12201199	a	t	++	1.51 (0.82 - 2.76)	0.182
3B	*TPMT*	rs12201199	a	t	++	1.52 (0.82 - 2.8)	0.183
2A	*TPMT*	rs12201199	a	t	-+	0.77 (0.4 - 1.48)	0.431
2B	*TPMT*	rs12201199	a	t	-+	0.77 (0.4 - 1.49)	0.435
1A	*TPMT*	rs12201199	a	t	-+	0.93 (0.53 - 1.61)	0.786
1B	*TPMT*	rs12201199	a	t	-+	0.94 (0.53 - 1.67)	0.825
4A	***TPMT***	**rs1800460**	**t**	**c**	**--**	**0.37 (0.14 - 0.94)**	**0.036**
4B	***TPMT***	**rs1800460**	**t**	**c**	**--**	**0.37 (0.15 - 0.94)**	**0.038**
3B	*TPMT*	rs1800460	t	c	--	0.52 (0.24 - 1.12)	0.094
3A	*TPMT*	rs1800460	t	c	--	0.53 (0.25 - 1.12)	0.096
1B	*TPMT*	rs1800460	t	c	+-	0.8 (0.38 - 1.7)	0.569
1A	*TPMT*	rs1800460	t	c	+-	0.86 (0.42 - 1.75)	0.681
2A	*TPMT*	rs1800460	t	c	+-	0.85 (0.38 - 1.91)	0.687

2B	*TPMT*	rs1800460	t	c	+-	0.85 (0.37 - 1.94)	0.701
1A	*TPMT*	rs1800462	Not available		??		
1B	*TPMT*	rs1800462	Not available		??		
2A	*TPMT*	rs1800462	Not available		??		
2B	*TPMT*	rs1800462	Not available		??		
3A	*TPMT*	rs1800462	Not available		??		
3B	*TPMT*	rs1800462	Not available		??		
4A	*TPMT*	rs1800462	Not available		??		
4B	*TPMT*	rs1800462	Not available		??		
2A	*WFS1*	rs62283056	c	g	++	1.29 (0.85 - 1.95)	0.23
2B	*WFS1*	rs62283056	c	g	++	1.28 (0.85 - 1.95)	0.24
1B	*WFS1*	rs62283056	c	g	++	1.14 (0.79 - 1.66)	0.474
3A	*WFS1*	rs62283056	c	g	+-	0.89 (0.61 - 1.3)	0.552
4A	*WFS1*	rs62283056	c	g	+-	1.14 (0.7 - 1.83)	0.604
1A	*WFS1*	rs62283056	c	g	-+	1.08 (0.76 - 1.54)	0.655
4B	*WFS1*	rs62283056	c	g	+-	1.11 (0.68 - 1.8)	0.683
3B	*WFS1*	rs62283056	c	g	+-	0.93 (0.63 - 1.38)	0.726

OR, odds ratio; CI, confidence interval.

[*] case-control designation and inclusion criteria of each analysis are specified in *Table S3*.

[1] Effect direction represents the effect direction in the GO-CAT cohort and the UK MAGIC cohort, respectively, where '+' means that allele 1 is linked to increased risk to develop the phenotype (OR > 1) and '–' to decreased risk (OR < 1). A question mark means this variant was absent in this cohort.
